# A Novel Technique for the Removal of an Intramedullary Femoral Guidewire Lodged in the Femoral Canal

**DOI:** 10.51894/001c.5785

**Published:** 2017-02-02

**Authors:** Conor Kasik, Michael Rosen, John Sauchak

**Affiliations:** 1 Department of Orthopaedic Surgery McLaren Greater Lansing; 2 Department of Osteopathic Surgical Specialties Michigan State University

**Keywords:** intramedullary fractures, fracture fixation, guide wires

## Abstract

Intramedullary nails are currently the most commonly used device for the fixation of intertrochanteric hip fractures. An initial threaded guidewire is used for localizing the site of entry and determining the final position of the fixation device. Hardware failure with guidewire breakage can complicate the procedure and lead to unplanned challenges for the surgeon. Predisposing mechanical properties of the hardware, along with technical surgical errors may lead to inadvertent breakage or migration of guidewires. The authors report a case of initial threaded starting guidewire migration into the femoral intramedullary canal with subsequent impaction into the distal femoral subchondral bone after advancement of the proximal femoral canal reamer. A method for antegrade removal of a lodged intramedullary guidewire through the distal femoral condyles is described. A set of key technical points to avoid this complication are also provided. Although guidewire migration during hip fracture surgery is a rare occurrence, it is important to recognize the technical measures that can be used to prevent this potentially devastating complication. Surgeons should be familiar with several different techniques for extraction of such hardware surrounding the hip, as there is no universally successful method.

## INTRODUCTION

Intramedullary fixation is the most commonly used construct for the current treatment of intertrochanteric hip fractures.^1^ Short intramedullary nails have become increasingly popular for the fixation of stable intertrochanteric hip fractures due to a shorter operative time, lower intraoperative blood loss, and a decreased need for blood transfusions when compared to the use of long intramedullary nails.^2^ These types of proximal femoral nailing systems utilize an initial threaded guidewire and cannulated reamer system to initially prepare the proximal canal.

During this procedure, a guidewire is used to determine the final intended position of the intramedullary nail. Failure to take preventative cautions along with technical surgical errors can lead to inadvertent hardware breakage or migration. Hardware failure with guidewire breakage can also complicate the procedure and lead to unplanned challenges to the surgeon. Many techniques have been previously described for extraction of broken Kirschner wires and guidewires, including extraction utilizing pituitary forceps, Kerrison rongeurs, and over-reaming the guidewire with a cannulated drill bit.^3–7^

In this paper, the authors report on a case of inadvertent migration of the initial 3.2 mm. distally threaded guidewire into the femoral intramedullary canal after use of the cannulated opening reamer. The guidewire became inadvertently impacted into the subchondral bone of the patient’s distal femur and was resistant to initial retrograde extraction techniques. Specific technical challenges presented during this case included the surgeons’ inability to provide a firm grip of the guidewire with the ongoing objective to perform a less invasive method of extraction in order to conserve the patient’s bone stock.

A specific method will be described for antegrade removal of an intramedullary femoral guidewire that has migrated into the femoral canal and become lodged in the distal femur. After strategic positioning of the patient’s leg, this technique can be generally reproduced using standard operating equipment.

### Description of Case

An 86-year-old female presented to the operating theatre with an Evan’s stable type intertrochanteric hip fracture after falling from a standing height. The decision was made to use a short trochanteric fixation nail (Synthes, West Chester, PA, USA).^8^ The patient was first placed supine on a standard radiolucent fracture table and initial closed reduction of the fracture was performed under fluoroscopic guidance.

A 3.2 millimeter (mm.) distally threaded guidewire that was 400 mm. in length was inserted using a standard greater trochanter starting point with entry slightly lateral to the tip of the greater trochanter. The guidewire was then advanced with a power drill down to the level of the lesser trochanter. The 17.0 mm. soft tissue protection sleeve and 3.2 mm. trocar were inserted over the guidewire down to bone, and a 17.0 mm. cannulated drill bit was used to ream the proximal femur.

There was initial resistance felt by the authors upon full advancement of the cannulated drill bit to the distal extent at the level of the lesser trochanter. After failure to fully advance the drill bit, fluoroscopic images were taken proximally to reveal the entire guidewire to be advanced into the intramedullary femoral canal.

**Figure 1:**
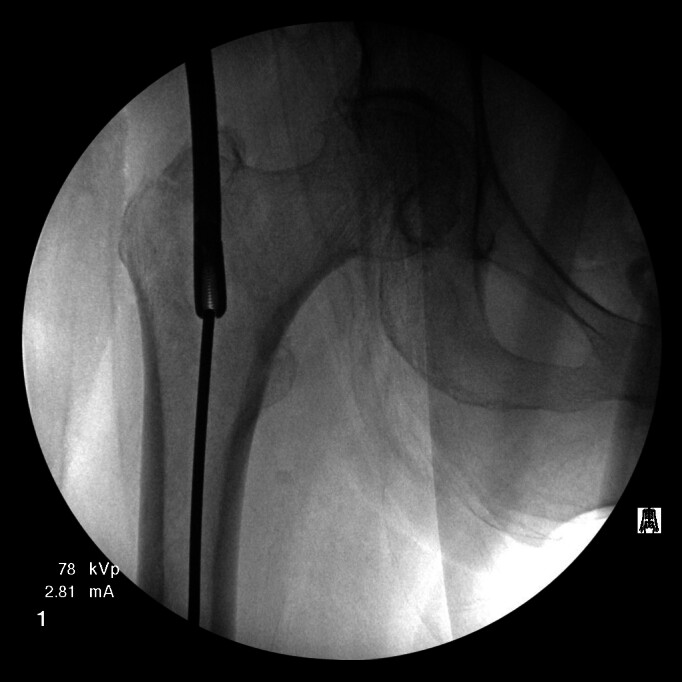
Attempted retrieval of threaded guidewire with pituitary rongeur.

**Figure 2:**
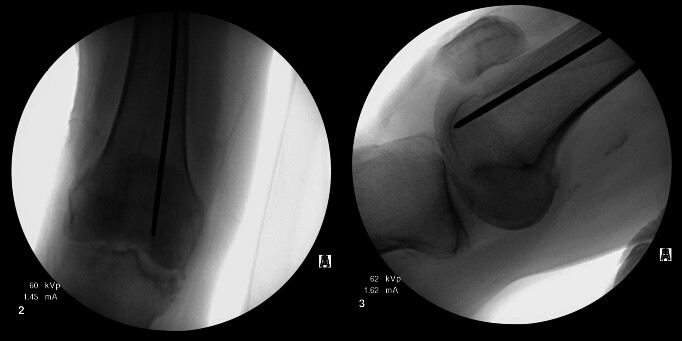
AP and lateral views of the right knee demonstrating distal subchondral impaction of the threaded guidewire.

Multiple failed attempts were made to remove the guidewire using Kocher forceps, arthroscopic basket forceps, needle drivers, and Kerrison rongeurs (see Figure 1). Additional fluoroscopic images of the distal femur were subsequently obtained, revealing the guidewire to be inadvertently impacted into the subchondral bone of the distal femoral condyle (see Figure 2). Due to the inability to retrieve the guidewire using other extraction methods, a decision was made by the surgeons to remove the guidewire antegrade through the distal femoral condyle.

**Figure 3:**
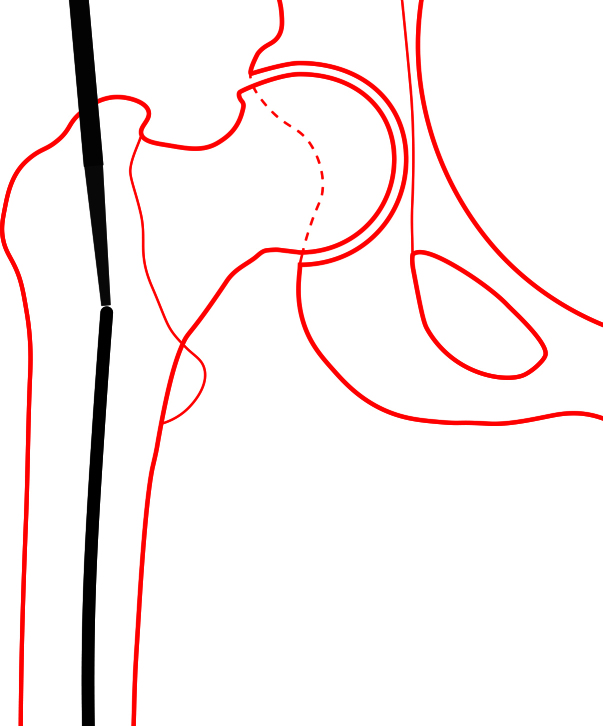
Schematic illustration demonstrating bone tamp placement into the opening of the proximal femur and in direct contact with the guidewire.

Traction on the operative leg was first released. The knee was then flexed to approximately 30° and held in the flexed position by a surgical assistant. A bone tamp was placed into the opening of the proximal femur and placed in direct contact with the guidewire (see Figure 3). A mallet was then used to gently tap the guidewire through the anterior aspect of the medial femoral condyle. The guidewire was advanced until tenting of the skin over the anterior knee was noted. A stab incision was made over the tip of the guidewire, and it was gently advanced further. Once enough guidewire was exposed, it was subsequently removed with a power drill.

## DISCUSSION

Orthopaedic hardware breakage or migration is a rare occurrence that can still pose major surgical challenges and result in potentially devastating effects. Guidewire complications during intramedullary nailing have been previously described in the past, with multiple case reports of complications due to migration into the thoracic and abdominal viscera.^3–7,9^ Recent literature concerning rates of orthopaedic hardware breakage has revealed that drill bits and Kirschner wires are the most commonly broken surgical instruments.^10^ Both mechanical and technical aspects of guidewire handling have been found to contribute to this phenomenon.^3–7^

Repeated use of guidewires for different orthopaedic patients can lead to an increased rate of breakage and failure, with rates approaching 0.18%.^3,10^ Mechanical deformation of the guidewire can also occur with repeated usage of the device, leading to both decreased bending and torsional strength of the wire.^5^ The results of a questionnaire survey administered by Ashford et al.^11^ to 248 orthopaedic units led to the proposal that surgeons should ensure single use by bending the guidewires after each case to prevent their re-use.

Technical errors by the surgeon can also predispose to an increased risk for inadvertent guidewire failure and breakage. Careful attention should therefore be paid to avoid inadvertently bending the guidewire or making any changes in its direction during insertion. Inserting the cannulated reamer over the guidewire with a different direction results in excessive contact stress upon the guidewire, possibly leading to migration and breakage.^7^

The cannulated drill bit should also be properly cleaned before reusing.^5^ Small cortical bony fragments may become incarcerated in the cannulated drill as it passes through the fracture site. The lumen of the drill bit may become blocked, leading to an increase in frictional force. This increased friction can result in bending or migration of the guidewire with forceful reaming. As the drill bit is in contract with the guidewire, an eccentric reaming point is created which can lead to guidewire breakage.^7^

This type of bony debris on the drill bit effectively decreases the diameter of the reamer, and prevents smooth gliding over the guidewire. ^3–7,9^ The reamer and guidewire then subsequently move as a single unit. With forceful undue reaming, the guidewire may be inadvertently propelled forward into the intramedullary canal.

Frequent fluoroscopic imaging utilizing both anteroposterior and lateral views should be employed when a cannulated instrument is not adequately progressing using powered instruments.^3–7,9^ Impediments to advancement of cannulated reamers include guidewire migration and eccentric bone reaming. Forceful reaming should therefore be avoided. Guidewire repositioning should be reassessed using fluoroscopic imaging if any resistance is felt during opening of the proximal femoral canal.

As indicated earlier in this paper, various techniques have been described to address removal of broken guidewires and Kirschner wires during hip fracture repair. These techniques include over-reaming with a cannulated drill bit, extraction using pituitary forceps or Kerrison rongeurs, arthroscopically assisted removal, and open arthrotomy with hip dislocation.^3–7^

The specific technical issues presented in this case included the inability to provide a firm grip of the guidewire, with the surgeons aiming to perform a lesser invasive method of extraction designed to conserve the patient’s bone stock. The method described by the authors in this paper involved use of a bone tamp and mallet to gently tap the guidewire through the distal femoral condyles.

Strategic positioning of the leg is critical in order to remove the guidewire through the femoral condyles. With traction on the foot released, the knee could be manipulated and flexed to approximately 30 degrees. This method provided an adequate exit angle for guidewire through the distal femoral condyles and can be performed with the use of standard operating room tools.

One disadvantage of this technique includes the potential damage to the articular cartilage of the knee. However, the small diameter footprint of the guidewire, in conjunction with its removal through the non-weightbearing aspect of the femoral condyles made these minor drawbacks that the authors were willing to accept to conserve this patient’s remaining bone stock.

Bone quality should also play a role in decision-making while utilizing this technique. The patient in the case had confirmed osteoporosis and decreased bone mineral density. The porosity of the patient’s bone made it feasible to tamp the terminally threaded guidewire out through the distal femur. Caution should be advised when employing this technique in younger patients, as their increased bone mass may prevent removal of the guidewire through the thickened bone of the femoral condyles.

## CONCLUSION

Although guidewire migration into the femoral intramedullary canal has not been well documented, it is important to recognize the technical and preventative aspects that can lead to this intraoperative complication.^9^ As outlined in Table 1, there are several key points in guidewire management that should be considered. Both preventive and proper technical techniques should be followed to decrease rates of guidewire migration and hardware breakage. Since no single surgical technique is universally successful for extraction of broken or migrated hardware, surgeons should be familiar with several different techniques that they can consider employing in varied hip fracture repair situations.

**Table 1.** Key points in guidewire management

1. Use guidewires only once. Be familiar with your institutions current practice regarding re-use. 2. Avoid inadvertent changes in direction when reaming over a guidewire. 3. Consider frequent intra-operative cleaning of the lumen of the cannulated reamer. 4. Perform both AP and Lateral imaging when failing to progress with the cannulated reamer. 5. Avoid forceful reaming and do not push through resistance. 6. Be familiar with several different techniques to remove broken guidewires.
